# Single Selenium Atomic Vacancy Enabled Efficient Visible-Light-Response Photocatalytic NO Reduction to NH_3_ on Janus WSSe Monolayer

**DOI:** 10.3390/molecules28072959

**Published:** 2023-03-26

**Authors:** Lin Ju, Xiao Tang, Yixin Zhang, Xiaoxi Li, Xiangzhen Cui, Gui Yang

**Affiliations:** 1 School of Physics and Electric Engineering, Anyang Normal University, Anyang 455000, China; 2 Institute of Materials Physics and Chemistry, College of Science, Nanjing Forestry University, Nanjing 210037, China; 3 School of Mechanical and Electrical Engineering, Chuzhou University, Chuzhou 239000, China

**Keywords:** NO reduction, WSSe monolayer, photocatalysis, density functional theory

## Abstract

The NO reduction reaction (NORR) toward NH_3_ is simultaneously emerging for both detrimental NO elimination and valuable NH_3_ synthesis. An efficient NORR generally requires a high degree of activation of the NO gas molecule from the catalyst, which calls for a powerful chemisorption. In this work, by means of first-principles calculations, we discovered that the NO gas molecule over the Janus WSSe monolayer might undergo a physical-to-chemical adsorption transition when Se vacancy is introduced. If the Se vacancy is able to work as the optimum adsorption site, then the interface’s transferred electron amounts are considerably increased, resulting in a clear electronic orbital hybridization between the adsorbate and substrate, promising excellent activity and selectivity for NORR. Additionally, the NN bond coupling and *N diffusion of NO molecules can be effectively suppressed by the confined space of Se vacancy defects, which enables the active site to have the superior NORR selectivity in the NH_3_ synthesis. Moreover, the photocatalytic NO-to-NH_3_ reaction is able to occur spontaneously under the potentials solely supplied by the photo-generated electrons. Our findings uncover a promising approach to derive high-efficiency photocatalysts for NO-to-NH_3_ conversion.

## 1. Introduction

A heightened consciousness of environmental and health issues has prompted significant endeavors to discover efficient and cost-effective technologies to detect, regulate, and remove a wide range of air pollutants, for example, nitric oxide (NO_x_), particulate matter (PM), and sulfur oxide (SO_x_). In this respect, NO, which is mainly emitted from the combustion of fossil fuels in stationary thermal power plants and internal combustion engines [1], is regarded as an essential threat to both human health and the global climate, given that it is a major factor in the formation of harmful photochemical smog, haze, and acid rain, etc. [2,3]. It has been reported that several methods, involving physical/chemical adsorption [4], heterogeneous catalytic reduction [5,6], and oxidation [7], have shown high efficiency in the selective sequestration and conversion of NO. However, such approaches have always been worked on especially for the treatment of NO in relatively high concentrations in the atmosphere, and both the capital and energy become unaffordable in the removal of NO at the ppb level. The development of an approach with the following characteristics is highly desirable but still challenging for practical ppb-level NO treating, i.e., significant NO conversion efficiency at room temperature, reliable performance in large-scale gas purification, and low-cost energy investment.

Moreover, in the field of NO conversion, where the N=O bond (204 KJ/mol) is more easily activated than the N≡N bond (941 KJ/mol), NH_3_ produced by electrocatalytic NO reduction reaction (NORR), as an attractive candidate for the traditional nitrogen reduction reaction (NRR), has recently been realized with many electrocatalysts [8,9,10]. Compared with the electrocatalytic process, the photocatalytic one is normally more attractive, since it is green, sustainable, and it inherits the advantages of natural photosynthesis. Though there are some achievements for the photocatalytic NRR that uses water as a proton source and reaction solvent [11,12,13,14,15,16,17], developing a stable and highly efficient photocatalyst for solar-driven NORR to NH_3_, to our knowledge, is still rarely reported.

Janus 2D transition metal dichalcogenides (TMD), an emerging 2D material that refers to layered materials with diverse surfaces, has recently sparked a lot of research interest in applications for photocatalytic energy conversion [18,19,20,21]. It is predicted that in Janus 2D TMD materials, the out-of-plane structural asymmetry-caused intrinsic dipole can manage the incompatible demands of the band gap between the high light-utilization rate and the capable redox capacity. Specifically, a sufficiently narrow band gap is usually required for high light utilization; however, a large band gap (≥1.23 eV) is usually required for a sufficient redox capability. With respect to two-dimensional polar photocatalysts, Yang et al. proposed that due to the presence of polarization, the top of the valence band and the bottom of the conduction band should be distributed on opposite sides, bringing about a potential difference that would improve the redox ability of the photoexcited carriers and reduce the requirement for a band gap [22]. Therefore, the Janus TMD materials are predicted to have a better photocatalytic property than the symmetrical traditional TMD materials [20,23]. Through replacing S atoms of WS_2_ with Se atoms with pulsed laser ablation plasmas [24] and hotting WSe_2_ and WS_2_ mixed powders under 1000 °C [25], the Janus WSSe monolayer, a classical Janus 2D TMD, has been successfully produced. Recently, Janus WSSe monolayers have also been reported to have significant applicability potential for photocatalytic overall water-splitting due to the excellent optical absorption, adequate redox capability, and high carrier separation [18].

Here, through density functional theory (DFT) calculations, we investigated NO adsorption upon 2H phase Janus WSSe monolayers, with and without manufactured Se-vacancy defects. The metallic 1T phase is not considered in our work, because it is typically unstable under ambient conditions [26]. To comprehend the coupling effect between NO and a substrate, a systematic explanation of the adsorption energy, charge density differential (CDD), and density of states (DOS) has been provided. We discovered that adding Se vacancy could lead to a shift in the physical-to-chemical nature of NO gas adsorption on the Janus WSSe monolayer, which could effectively activate NO gas molecules, thus making the NORR possible. Then, we investigated the photocatalytic NORR on the defective Janus WSSe monolayer, by estimating the optical absorption, redox capacity, and the driving force of the photo-exited electron for NORR. At last, we discuss the competition between the NORR and hydrogen evolution reaction (HER). Our results reveal that the defective WSSe with the outstanding photocatalytic NORR performance could be used as a novel platform for NO conversion.

## 2. Results and Discussion

### 2.1. NO Physical Absorption upon a Pristine Janus WSSe Monolayer

#### 2.1.1. Adsorbing Site Selection and $E_{ads}$

**Figure 1:**
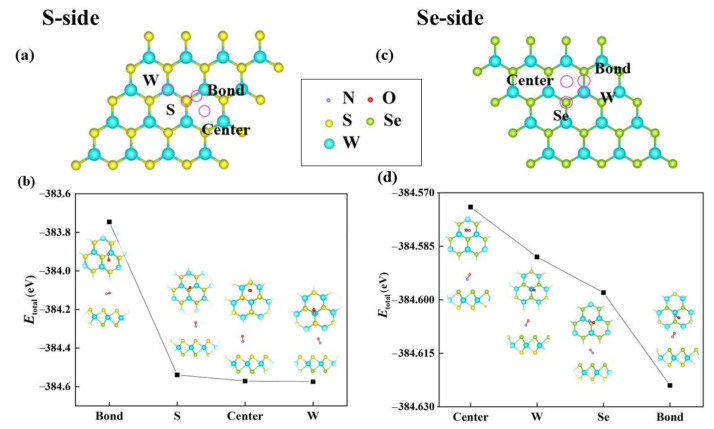
(**a**) S-side and (**c**) Se-side possible adsorption sites (symbolized in purple circles) considered for the case of pristine WSSe. The $E_{total}$ of (**b**) S-side and (**d**) Se-side NO adsorption systems with various adsorption sites. The N, O, S, Se, and W atoms are represented with the purple, red, yellow, green, and blue balls, respectively, and this color scheme is also used in Figures 2, 4, 5, 7 and 8.

A W layer is sandwiched between the S and Se layers to form the Janus WSSe single layer. Like WSe_2_ or WS_2_, the matrix materials, the Janus WSSe monolayer has a honeycomb structure [27]. According to calculations, Janus WSSes lattice constant is 3.26 Å, which is in the middle of the range between WS_2_ and WSe_2_, which are its parent materials. It is highly desirable to investigate whether the vertical intrinsic dipole that is brought on by the asymmetric structure will enhance the gas sensing properties of Janus WSSe, similar to how it did in the case of Janus MoSSe [28]. In this work, geometric properties of NO adsorption on both sides of Janus WSSe were initially taken into consideration, as seen in Figure 1a,c. Every adsorption situation involves placing one NO molecule upon a WSSe monolayer’s 4 × 4 supercell whereas the system is fully relaxed. In addition, several potential adsorption sites have been taken into account, i.e., the top sites over the W-Se/(W-S) bond (named **Bond**), S/Se/W atom (named **W/Se/S**), and hexagon’s center (named **Center**).

In accordance with Equation (1), it is observed that $E_{total}$ dominates $E_{ads}$, since $E_{sub}$ and $E_{gas}$ are invariant at the various adsorption sites. Herewith, to explore the most stable adsorption configuration, we calculated the $E_{total}$ of the NO adsorption in both the S-side and Se-side patterns by considering various adsorption sites. For the NO adsorption on the S-side, as shown in Figure 1b, we found that $E_{total}$ achieves the minimum (−384.57 eV) if it lies on the **W** site, denoting the most stable adsorption conformation. For the Se-side NO adsorption, once the molecule was placed over the **Bond** site, the system had the lowest $E_{total}$ (−384.62 eV), implying the steadiest adsorption site, as shown in Figure 1d. Additionally, the Se-side steadiest adsorption system $E_{total}$ is lower than the S-side one by 0.05 eV, leading to the conclusion that NO gas molecules are more likely to adsorb in the Se plane. Consequently, we chose the Se-side NO adsorption on the **Bond** site to stand for the case of NO gas molecule adsorbing on the pristine Janus WSSe monolayer. The absolute value of $E_{ads}$ for a physisorption is often less than 1 eV [29,30,31,32]. Therefore, the adsorption for the configuration is most likely a physisorption with the $E_{ads}$ equal to −0.21 eV. This will be discussed in the following section for further investigations of the physisorption.

#### 2.1.2. Adsorption Mechanism

**Figure 2:**
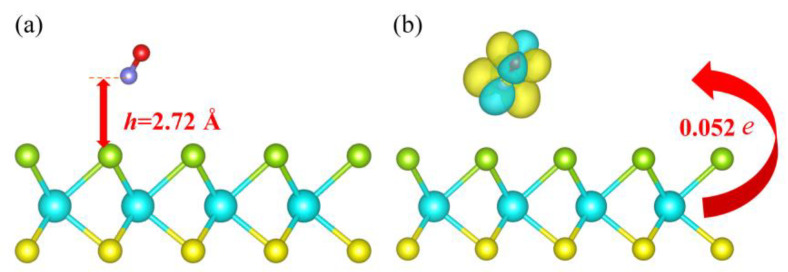
The optimized structure in a side view (**a**) and CDD image (**b**) for adsorption system, composed of pristine Janus WSSe monolayer and NO gas molecule. The *h* in red represents the adsorption distance from NO to pristine Janus WSSe monolayer. Charge deposition (exhaustion) is indicated by yellow (cyan) areas. The value of the isosurface is 0.002 *e* Å^−3^, and that red arrow indicates the charge transfer direction.

The NO molecule kept parallel to the surface of the substrate after adsorption at a vertical distance away of 2.72 Å as its nitrogen atoms tended to the surface, as seen in Figure 2a. Additionally, the smallest distance from the NO molecule’s N atom to its nearest Se atom reaches 3.53 Å, which is significantly longer than the Se-N bond’s length of 1.81Å. Moreover, as shown in Figure 2b, the CDD results indicate that the charge redistribution mainly takes place at the NO gas molecule, and only very rare electrons (merely 0.052 *e*) migrate from the substrate to the NO molecule, resulting in the weak interaction between them.

**Figure 3:**
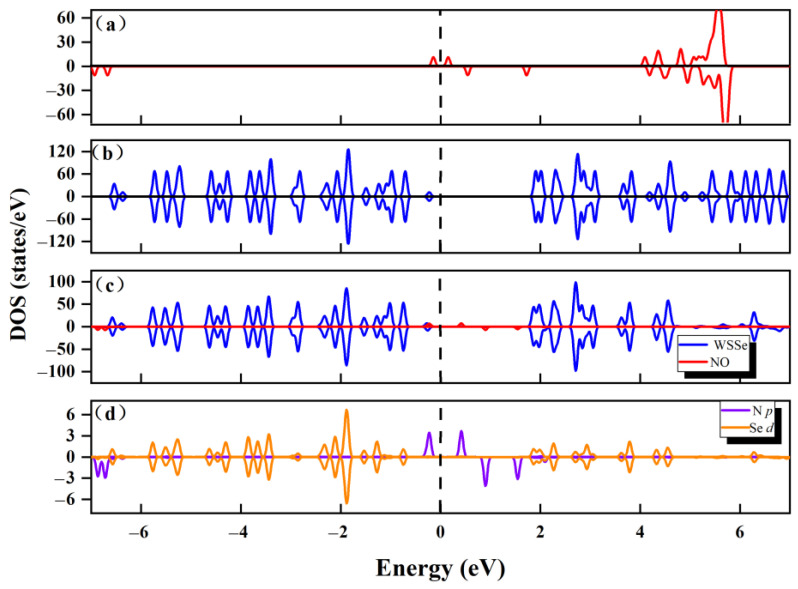
Total DOS of (**a**) a free NO molecule, as well as (**b**) a pure pristine WSSe. (**c**) The adsorption system’s partial DOS. Dark blue is used to represent the WSSe component while red is used to represent the NO portion. (**d**) N *p* orbitals (marked in purple) of the adsorbed NO and Se *p* orbitals (marked in orange) of the Se atoms most near adsorbed NO molecule. The Fermi level is indicated by the perpendicular dashed line.

This adsorption configuration’s pertinent DOS has been computed. Figure 3a–c shows how little the gas molecule as well as the monolayer were altered after adsorption, with respect to DOS. This is consistent with the minute interface transfer electron, which suggests that neither the electronic properties of WSSe nor NO have changed noticeably. The very weak connection seen between the WSSe monolayer and NO is shown by their poor orbital hybridization, which is consistent with the previous statement. Additionally, as shown in Figure 3d, the Se *p* orbital from the Se atom in the WSSe monolayer, most near the NO gas molecule, as well as the N *p* orbital from the N atom in the NO gas molecule, are independent of one another. Based on the investigation above, the NO adsorption over the pristine WSSe should be physisorption.

### 2.2. NO Chemisorption and Reduction Reaction over Defective Janus WSSe Monolayer

NO physisorption on pristine WSSe is suitable for use in gas collection systems. Yet, the need for NO chemisorption is greater when it comes to treating gases or accelerating chemical reactions, which calls for a substrate with a stronger adsorption capability. Based on earlier pertinent findings, it is found that adding a few vacancy defects might affect the electrical property and hence significantly increase the stability of specific geometric formations [33,34]. Therefore, we create vacancy defects in the Janus WSSe monolayer in an effort to increase NO gas molecule adsorption. Here, we concentrate on the single Se vacancy defect for the following three reasons: (I) The Se vacancy is easier to form than other kinds of vacancy defects at the Janus TMD monolayer due to its relatively lower formation energy [35]. (Ⅱ) As previously mentioned, NO gas molecules tend to adsorb on the Se-side of the pristine monolayer. (Ⅲ) Photo-reduction has been theoretically demonstrated to take place on the Se-side of the pristine monolayer [18], which shows a potential for the NORR to NH_3_.

#### 2.2.1. Adsorbing Site Selection and $E_{ads}$

**Figure 4:**
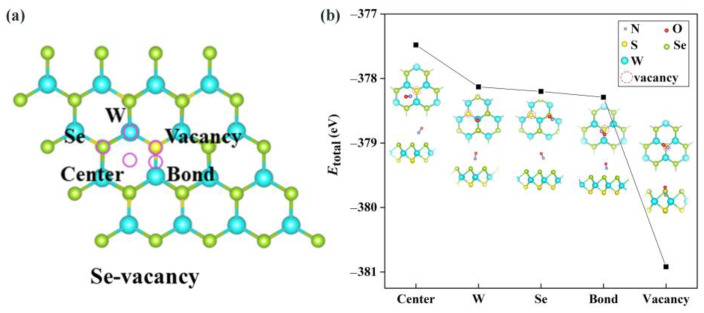
(**a**) Possible adsorption sites (symbolized in purple circles) considered for the case of defective WSSe. (**b**) The $E_{total}$ of this NO adsorption system with various adsorption sites.

As depicted in Figure 4a, for the defective WSSe monolayer, five possible adsorption sites were taken into consideration. They are the **Center** (the top site above the center of the hexagon), **W** and **Se** (the top of the W and Se atoms, separately), **Bond** (the top site above the W-Se bond), and **Vacancy** (the top site above the Se vacancy defect) adsorption sites. The adsorption system $E_{total}$ was employed to capture the most likely adsorption morphology, analogous to the pristine monolayer situation. The $E_{total}$ was minimized when NO was adsorbed on the **Vacancy** site (see Figure 4b), so the **Vacancy** site is the most likely adsorption site in this case. The $E_{ads}$ under the condition is −2.92 eV, which represents an order of magnitude that is more negative than that on pristine WSSe (−0.21 eV). It is clear that the introduction of Se vacancies resulted in an effective enhancement of the NO adsorption. From the anomalously negative $E_{ads}$, it can be tentatively determined that this NO adsorption on defective WSSe is chemisorption. We explore this issue in more depth in the next section.

#### 2.2.2. Adsorption Mechanism

**Figure 5:**
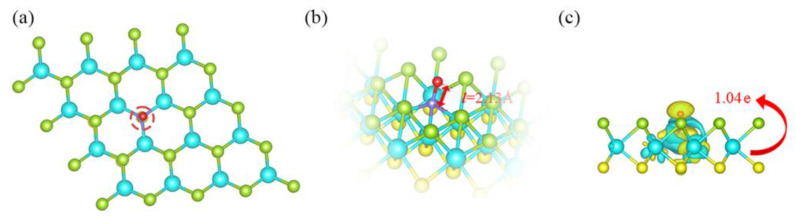
(**a**) Top, (**b**) side, and (**c**) CDD images of the optimal structures for the adsorption system under the defective WSSe case. The *l* in red stands for N-O bond length. Charge accumulation (depletion) is indicated by areas that are yellow (cyan). The value of the isosurface is 0.002 e Å^−3^. The red number in CDD image indicates how much charge transferred from the substrate to the molecule.

The nitrogen atom in the N-O bond of the NO gas molecule takes a nearly vertical orientation, as seen in Figure 5a, pointing to the surface of the monolayer. At the surface of the monolayer, the nitrogen atom connects with the three tungsten atoms that are next to it. As a result, the adsorption is unquestionably chemisorption, which is consistent with that outcome produced by its adsorption energy as stated before. Additionally, we evaluated the N-O bond length for quantitatively analyzing how the morphology of NO changed pre and post adsorption. Before adsorption, it is 1.17 Å, and as shown in Figure 5b, it stretches to 2.13 Å post adsorption, indicating electron redistribution in NO through the adsorption process. A large number of electrons (1.04 *e*) move to the adsorbate from the damaged Janus WSSe layer, as can be observed in Figure 5c, where there are notable charge redistributions in the adsorption system. For gas sensors, resistivity fluctuation is typically brought on by adsorption-induced charge transfer, which is a crucial indicator of sensing merits and could be determined by experiments [36,37].

**Figure 6:**
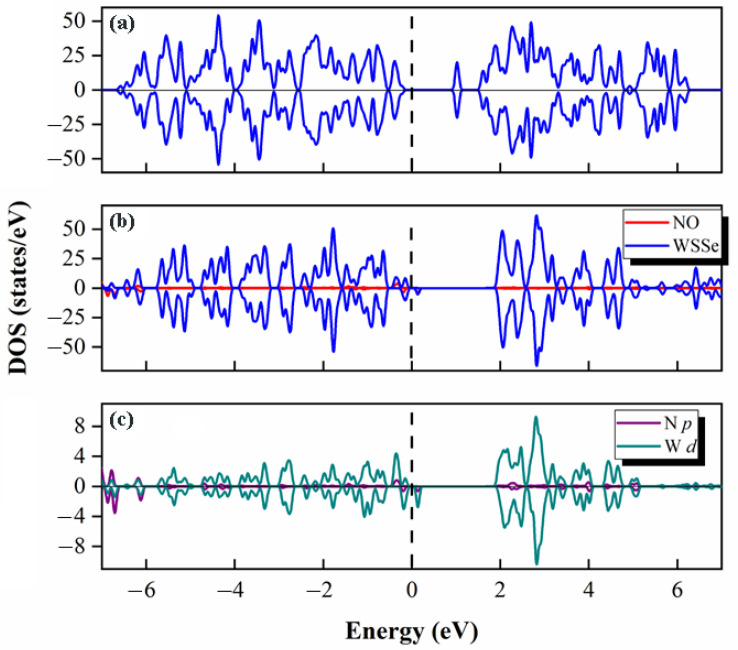
(**a**) Total DOS of the defective WSSe without NO adsorption. (**b**) The adsorption system’s partial DOS. Dark blue indicates the WSSe component, whereas red indicates the NO portion (the enlarged view is shown in Figure S1). (**c**) The N *p* orbitals from the adsorbed NO gas molecule (marked in dark purple) and the W *d* (marked in dark green) orbitals of these three W atoms that are attaching to the N atom from NO. The Fermi level is indicated by the vertical dashed line.

We compute the pertinent DOS and display them in Figure 6 to obtain a greater understanding of the electronic characteristics for this chemisorption system. The two parts of the chemisorption system have a strong electronic orbital hybridization (see Figure 6b). This demonstrates that they interact strongly, which accounts for the observation that NO was closely bound to the substrate. Additionally, the coupling between the N *p* orbital from NO and W *d* orbitals of the W atoms, which bond to the N atom of NO, contributes significantly to the interaction (see Figure 6c). The comparison of the DOS of NO gas molecules between pre and post adsorption (see Figure 3a and Figure S1) shows that the DOS is significantly delocalized after adsorption, indicating a sharp electron redistribution in NO, which is responsible for the visual N-O bond shift. From these results, we further demonstrate that the NO adsorption over the defective WSSe monolayer is chemisorption. Additionally, adding Se vacancies into Janus WSSe can wondrously trigger the NO physisorption-to-chemisorption transition.

#### 2.2.3. Photocatalytic NORR

The obvious N-O bond elongation of the NO gas molecule caused by adsorption indicates that this molecule is activated, thus making the further NORR possible. Since the defective Janus WSSe monolayer is semiconductor with a bandgap of 1.22 eV (see Figure 6a), which is not suitable to act as an electrocatalysts, we study the photocatalytic NORR on the defective WSSe next.

**Figure 7:**
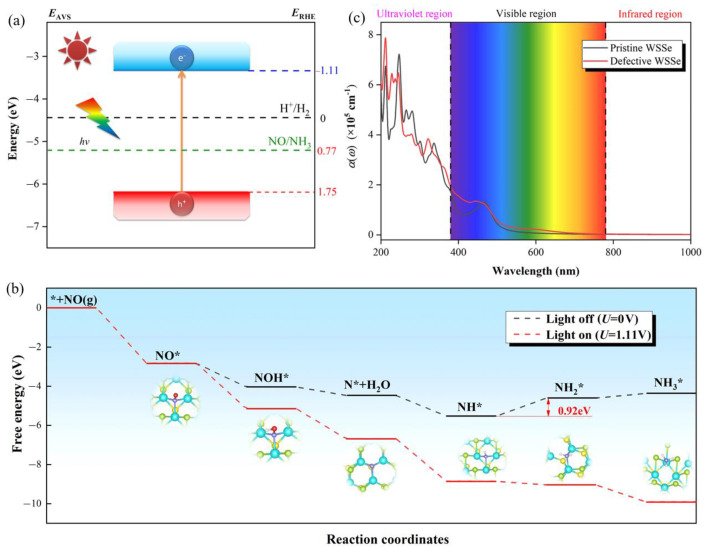
(**a**) A schematic representation of the Janus WSSe monolayer’s band edge positions in relation to the reversible hydrogen electrode (RHE) at pH = 0. * stands for the adsorption site at the surface of catalyst. Relative energy levels to the absolute vacuum scale (AVS) and RHE are represented by *E*_AVS_ and *E*_RHE_. (**b**) Gibbs free energy diagrams of NORR to NH_3_ on defective Janus WSSe monolayer under *U* = 0 and *U* = 1.11 V. The applied potential that a photo-excited electron provides is *U* = 1.11 V. (**c**) The Janus WSSe monolayer’s optical absorbance in both pristine and defective states.

The band edges of a semiconductor must line up with the potentials of the redox half-reactions in order for it to be active for the NO photo-reduction. Whether the photo-catalytic NORR can proceed spontaneously depends directly on the strength of the external potentials that are provided by the photo-generated carriers [38]. The energy difference between the electron acceptor states and the hydrogen reduction potential (H^+^/H_2_) and the potential of photogenerated electrons for NORR (*U_e_*) (Figure 7a) has been reported to be −1.11 V for the Janus WSSe monolayer at pH = 0 [18], which is significantly more negative than the theoretical potential of NORR (0.77 V vs. RHE [39]). A good resistance to photoinduced corrosion is facilitated by high *U_e_*, which denotes the fact that photogenerated electrons of the Janus WSSe monolayer would prefer to be transferred to react with H^+^ rather than with themselves [38,40].

There are five proton-coupled electron transfer steps during the NORR to NH_3_ process (NO + 5H^+^ + 5e^−^ →NH_3_ + H_2_O). The free energy diagram as well as the related intermediate products for the NORR to NH_3_ on the defective Janus WSSe monolayer are given in Figure 7b. The most favorable path is NO*→NOH*→N*→NH*→NH_2_*→NH_3_*. We can see that the electrocatalytic steps, including NO*→NOH*, NOH*→N*, and *N→*NH, are exothermic by −1.21, −0.44, and −1.05 eV, respectively. The third electrocatalytic step, i.e., N*+ e^−^ + H^+^ → NH*, means that one H atom adsorbs on the N* to form NH*. In order to explore the ease of the NH* formation, we add the detailed analysis on the interaction between the H atom and N atom in the NH* based on the partial DOS. As shown in Figure S3, there is an obvious hybridization between N *p* and H *s* orbitals near the Fermi level, causing a strong attraction to each other. Therefore, the H atom could easily adsorb on the N*, making the reaction of NH* formation exothermic. Moreover, the exothermic reaction of NH* formation from hydrogenating N* on various electro-/photo-catalysts has been reported [6,41,42,43]. The other electrocatalytic steps, i.e., NH*→NH_2_* and NH_2_*→NH_3_*, are endothermic separately, with free energy uphills of 0.92 and 0.24 eV. Excitingly, all NORR intermediate processes become exothermic when taking into account the external potential provided by photo-excited electrons (*U* = 1.11 V), demonstrating the spontaneous NORR with lighting (red line in Figure 7b).

An efficient photocatalyst must have a high photoconversion efficiency in order to start the photocatalytic conversion of NO to NH_3_. Due to the narrowed direct band gap (see Figure S2), notably, Se vacancy introduction on the Janus WSSe monolayer leads to a redshift of the initial optical absorption peak (at 600.45 nm, red line), which is relative to the baseline value from the pristine Janus WSSe monolayer (at 466.28 nm, black line); therefore, it expands the optical absorption into visible regions, as shown in Figure 7c. Moreover, notably, the biggest absorption peak of the defective Janus WSSe monolayer among visible region, reaches up to 1.35 × 10^5^ cm^−1^ (at 444.42 nm, red line), exceeding the one of pristine Janus WSSe monolayer (1.30 × 10^5^ cm^−1^ at 466.28 nm, black line), which is comparable to some other photocatalysts, such as MoSSe–GaN (2.74×10^5^ cm^−1^ at 425 nm) [44], MoSSe–AlN (3.95 × 10^5^ cm^−1^ at 412 nm) [44], and graphene–MoSSe (about 4.00 × 10^5^ cm^−1^ at 500 nm) [45]. The broadened optical absorption region and elevated optical absorption peak reveal that photons within a wider energy range can be utilized by bringing in the Se vacancy defect in the Janus WSSe monolayer.

#### 2.2.4. Selectivity for NORR vs. HER

**Figure 8:**
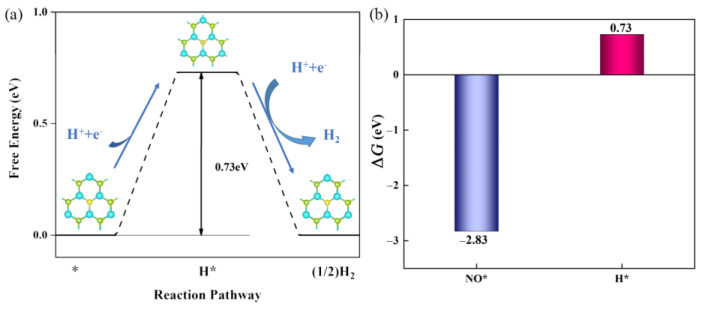
(**a**) Gibbs free energy diagram of HER on defective Janus WSSe monolayer. (**b**) Δ*G*_NO*_ vs. Δ*G*_H*_ of defective Janus WSSe monolayer.

By depleting proton–electron pairs out of an electrolyte solution, the substantial competitive side reaction known as the hydrogen evolution reaction (HER) may drastically reduce the faradaic efficiency of NORR [46,47]. According to the Brønsted–Evans–Polanyi relation [48,49], lower Δ*G* reactions have lower reaction barriers and are therefore kinetically more preferred. Hereby, as shown in Figure 8a, we calculated the Gibbs free energy difference of H* formation (Δ*G*_H*_), and compared it with the one of NO* formation (Δ*G*_NO*_). The **Vacancy** site is the most feasible adsorption site for single H atom in defective Janus WSSe (more details of screening process can be found in the supporting information). As displayed in Figure 8b, Δ*G*_NO*_ (−2.83 eV) is much lower than Δ*G*_H*_ (0.73 eV), indicating that the active sites in the defective Janus WSSe monolayer will be preferentially occupied by *NO. According to the previous method used to judge the selectivity between HER and NORR [50], we could draw a conclusion that, NORR is highly preferred over HER.

#### 2.2.5. Selectivity for NO-to-NH_3_ Pathway vs. Other NORR Pathways

Besides HER, some other side reactions, such as the formation of N_2_O_2_ and N_2_, perhaps restrain the production of NH_3_ as well, so the selectivity of the reaction pathways for NORR should be considered. As mentioned before, due to spatial confinement, NO molecules can only ever assume the end-on orientation due to the N atom coupled with the exposed metal sites when adsorbing on the defective Janus WSSe monolayer. The reaction can only take place through the distal channel according to this NO adsorption model [51,52]. Most crucially, the Se vacancy defect’s constrained space will successfully block the approach of two NO molecules, preventing the formation of N_2_O_2_, and N atoms can be firmly bound by the under-coordinated active sites in the vacancy to obstruct *N diffusion. Hence, N_2_ production is excluded as a result of spatial constriction. Hence, there is a high selectivity of the NO-to-NH_3_ reaction pathway guaranteed at a defective WSSe.

## 3. Materials and Methods

In this study, all the DFT simulations are operated with the Vienna Ab initio Simulation Program software package (Hanger team, University of Vienna, version 5.3) [53,54]. The exchange–correlation energy was simulated using the generalized gradient approximation of Perdew–Burke–Ernzerhof. We utilize the zero-damped DFT-D3 approach suggested by Grimme [55] to characterize the van der Waals (vdW) force. The plane wave basis set’s cutoff energy was determined to be 500 eV. With a fixed lattice constant, all the internal coordinates were let to relax throughout optimization. As NO is a paramagnetic molecule, spin polarization is used when calculating the NO adsorption [56]. The computational model is built by one NO gas molecule adsorbing on a 4 × 4 supercell of pristine/defective Janus WSSe monolayer. Employing a 2 × 2 × 1 K point sampling, the Brillouin zone was sampled for integration using the Monkhorst-Pack technique [57] for structural optimization and electronic properties computations. To minimize the impact of interlayer contact, a 30 Å space was given down the direction that is normal to the plane. Moreover, the calculation of Gibbs free energy change for NORR is operated with the computational hydrogen electrode (CHE) model [58], and the solvent effect is considered with the implicit solvent model implemented in VASPsol [59,60]. More simulation details of the Gibbs free energy can be found in the supporting information.

The following formula is used to calculate NOs adsorption energy ($E_{ads}$) on both the damaged and unaltered WSSe monolayer [61,62],

(1) $$E_{ads}=E_{total}-E_{sub}-E_{gas}$$

where $E_{sub}$ and $E_{gas}$ separately are the clean substrate (pristine/defective Janus WSSe monolayer) and the sole NO molecule total energies, while $E_{total}$ is the adsorption system total energy. An exothermic adsorption is indicated by a negative value for $E_{ads}$. The strength of the gas adsorption increases as $E_{ads}$ is more negative.

The following equation was used to carry out the plane-integrated CDD,

(2) $$∆\rho =\rho_{total}-\rho_{sub}-\rho_{gas}$$

where $\rho_{gas}$ and $\rho_{sub}$ independently represent the charge density of the NO molecule and substrate, meanwhile, $\rho_{total}$ is the adsorption system charge density.

The absorption coefficient $a\left(\omega \right)$ to assess the ability of sunlight harvesting is calculated following the formula below [63],

(3) $$a\left(\omega \right)=\sqrt{2}\frac{\omega }{c}{\left(\sqrt{\varepsilon_{1}{\left(\omega \right)}^{2}+\varepsilon_{2}{\left(\omega \right)}^{2}}-\varepsilon_{1}\left(\omega \right)\right)}^{\frac{1}{2}}$$

where the real and imaginary components of a frequency-dependent dielectric function are denoted by *ε*_1_ and *ε*_2_, respectively, while the vacuum speed of light is denoted by *c*.

## 4. Conclusions

In our work, the NO adsorption on both pristine and defective WSSe monolayers has been theoretically investigated. On the pristine WSSe monolayer, the NO adsorption is physisorption based on minor adsorption energy, a large adsorption distance, and feeble electron orbital hybridization. By adding Se vacancies to WSSe, it is possible to convert the NO physisorption into chemisorption by significantly increasing the amount of interfacially transferred electrons and inducing significant electronic orbital coupling between the two components of the adsorption system. The powerful NO chemisorption gives defective WSSe high activity and selectivity for NORR. The active site has strong NORR selectivity for NH_3_ production because the limited area of the Se vacancy defect may efficiently hinder the N-N bond coupling of NO molecules and the *N diffusion. Moreover, the potential provided by photogenerated electrons in the defective Janus WSSe monolayer is sufficient to drive a spontaneous NORR to NH_3_. Our findings suggest an energy-saving and environmentally friendly strategy for direct NO-to-NH_3_ conversion, which is anticipated to spur greater investigation into photocatalysts for NO-to-NH_3_ conversion.

## Data Availability

The data presented in this study are available in Supplementary Materials.

## Supplementary Materials

The following supporting information can be downloaded at: https://www.mdpi.com/article/10.3390/molecules28072959/s1, Figure S1: The enlarged view for the partial density of states of NO portion from the adsorption system; Figure S2: The band structures of the pristine and defective Janus WSSe monolayers; Figure S3: The N *p* orbitals and the H *s* orbitals of intermediate NH*; Figure S4: Top view of the optimal structures for H* with H atom on **Center** and **Se** sites in the defective WSSe monolayer; Table S1: The total energy of H* with H atom on different deposition sites; Screening adsorption site for single H atom in defective Janus WSSe; Free energy difference in NORR.
