# Supplementary material for: Single Selenium Atomic Vacancy Enabled Efficient Visible-Light-Response Photocatalytic NO Reduction to NH3 on Janus WSSe Monolayer
Source: Molecules. 2023 Mar 26;28(7):2959. doi: 10.3390/molecules28072959 (PMC10095809; doi:10.3390/molecules28072959)
Supplement: Supplementary file 1 [file molecules-28-02959-s001.zip › molecules-2250449-supplementary.pdf]

Single Selenium Atomic Vacancy Enabled Efficient Visible-Light-Response Photocatalytic NO Reduction to NH_3 on Janus WSSe Monolayer

Lin Ju[1,*], Xiao Tang[2], Yixin Zhang[1], Xiaoxi Li[1], Xiangzhen Cui[1], and Gui Yang[3,*]

[1]School of Physics and Electric Engineering, Anyang Normal University, Anyang, 455000, China

[2]College of Science, Nanjing Forestry University, Nanjing, 210037 P. R. China

[3]School of Mechanical and Electrical Engineering, Chuzhou University, Chuzhou 239000, China

Corresponding Email: julin@aynu.edu.cn (L.J.); fengmingchun@chzu.edu.cn (G.Y.)

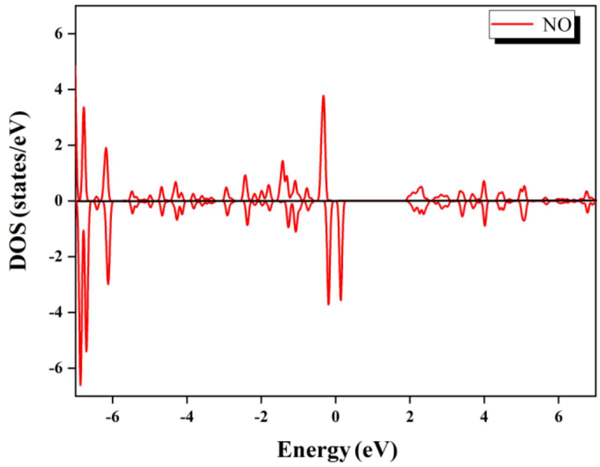

Figure S1. The enlarged view for the partial density of states of NO portion from the adsorption system (NO gas molecule adsorbed on defective Janus WSSe monolayer).

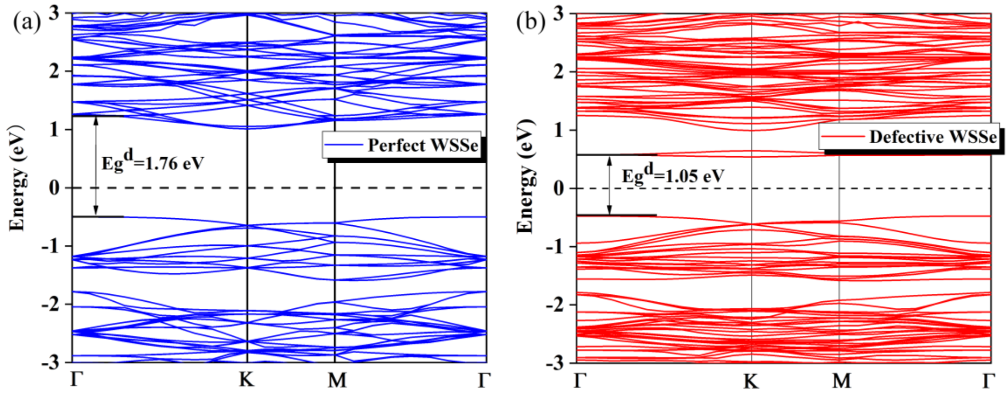

Figure S2. The band structures of the (a) pristine and (b) defective Janus WSSe monolayers. The direct band gap (Eg^d) for them have been denoted, respectively.

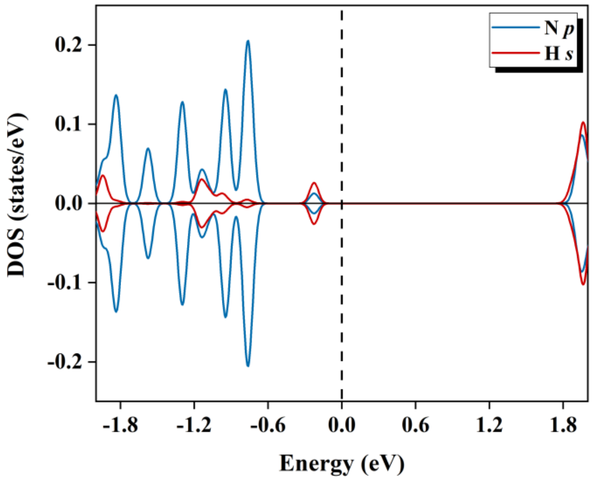

Figure S3. The N *p* orbitals (marked in blue) and the H *s* (marked in red) orbitals of intermediate NH*. The Fermi level is indicated by the vertical dashed line.

Screening adsorption site for single H atom in defective Janus WSSe

Similar to the case of NO adsorption, as shown in Figure 4a, we considered five adsorption sites for the H atom adsorption, namely, **W**, **Bond**, **Vacancy**, **Center** and **Se**. As displayed in Table S1, after relaxation, the H atom moves to the same position (**Vacancy**), when it is separately placed at the **W**, **Bond** and **Vacancy** sites at the initial state. The optimized structures for H* with the **Vacancy**, **Center** and **Se** as adsorption sites in the final state are shown in Figures 8a, S4a, and S4b. The total energy for the adsorption system with the **Vacancy** adsorption site is obviously lower than the ones with **Center** and **Se** adsorption sites. Therefore, the **Vacancy** site is the most feasible adsorption site for single H atom in defective Janus WSSe.

Table S1 The total energy of H* with H atom on different deposition sites of Figure 4a.

Before relaxation	After relaxation	Total energy (eV)
**W**	**Vacancy** (see Figure 8a)	-368.63
**Bond**		
**Vacancy**		
**Center**	**Center** (see Figure S4a)	-366.50
**Se**	**Se** (see Figure S4b)	-366.66

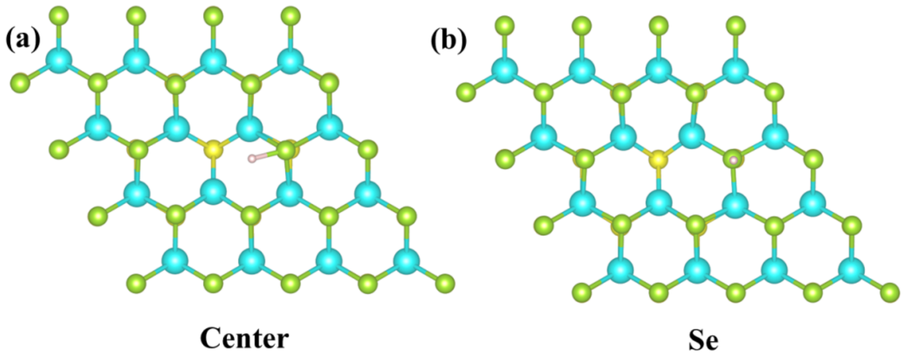

Figure S4. Top view of the optimal structures for H* with H atom on (a) **Center** and (b) **Se** sites in the defective WSSe monolayer.

Free energy difference in NORR

Free energy difference (ΔG) in the water redox reactions is calculated according the approach proposed by Nørskovet *al.*[1] The formula at pH = 0 without solar irradiation can be defined as below (equation S1):

$$\Delta G = \Delta E + \Delta E_{\mathrm{zpe}} - T\Delta S \tag{S1}$$

where ΔE is the adsorption energy, ΔE_{zpe} and ΔS are the difference in zero point energy and entropy difference between the adsorbed state and the gas phase, respectively. Table S2 presents E_{zpe} and TS (at 298.15 K) of the free molecules and the adsorbed species along the most favourable reaction pathway for NO-to-NH_3 reduction reaction happened on defective Janus WSSe monolayer.

There are five steps to transform NO into NH_3 molecule in the NO reduction reaction (NORR) along the minimum energy path, which can be written as:

$$\mathrm{NO}* + \mathrm{H}^+ + \mathrm{e}^- \rightarrow \mathrm{NOH}* \tag{Sa}$$

$$\mathrm{NOH}* + \mathrm{H}^+ + \mathrm{e}^- \rightarrow \mathrm{N}* + \mathrm{H_2O} \tag{Sb}$$

$$\mathrm{N}* + \mathrm{H}^+ + \mathrm{e}^- \rightarrow \mathrm{NH}* \tag{Sc}$$

$$\mathrm{NH}* + \mathrm{H}^+ + \mathrm{e}^- \rightarrow \mathrm{NH_2}* \tag{Sd}$$

$$\mathrm{NH_2}* + \mathrm{H}^+ + \mathrm{e}^- \rightarrow \mathrm{NH_3}* \tag{Se}$$

where * means the adsorbed materials, NOH*, N*, NH*, NH_2* and NH_3* represent the adsorbed intermediates.

For each reaction of NORR, the free energy difference under the effect of pH and an extra potential bias can be written as:

$$\Delta G_{\mathrm{Sa}} = \Delta G_{\mathrm{NOH}*} = G_{\mathrm{NOH}*} - \frac{1}{2}G_{\mathrm{H_2}} - G_{\mathrm{NO}*} + \Delta G_U + \Delta G_{pH} \tag{S2}$$

$$\Delta G_{\mathrm{Sb}} = \Delta G_{\mathrm{N}*} = G_{\mathrm{N}*} + \frac{1}{2}G_{\mathrm{H_2O}} - G_{\mathrm{NOH}*} - \frac{1}{2}G_{\mathrm{H_2}} + \Delta G_U + \Delta G_{pH} \tag{S3}$$

$$\Delta G_{\mathrm{Sc}} = \Delta G_{\mathrm{NH}*} = G_{\mathrm{NH}*} - \frac{1}{2} G_{H_2} - G_{\mathrm{N}*} + \Delta G_U + \Delta G_{pH} \qquad (S4)$$

$$\Delta G_{\mathrm{Sd}} = \Delta G_{\mathrm{NH_2}*} = G_{*\mathrm{NH_2}} - \frac{1}{2} G_{H_2} - G_{\mathrm{NH}*} + \Delta G_U + \Delta G_{pH} \qquad (S5)$$

$$\Delta G_{\mathrm{Se}} = \Delta G_{\mathrm{NH_3}*} = G_{\mathrm{NH_3}*} - \frac{1}{2} G_{H_2} - G_{\mathrm{NH_2}*} + \Delta G_U + \Delta G_{pH} \qquad (S6)$$

Where ΔG_{pH} ($\Delta G_{pH} = \mathrm{k_B} T \times ln10 \times pH$) represents the free energy contributed in different pH concentration. ΔG_U ($\Delta G_U = -eU$) denotes extra potential bias provided by an electron in the electrode, where U is the electrode potential relative to the standard hydrogen electrode (SHE). The catalytic activity was evaluated by the theoretical limiting potentials, U_l (U_l = -ΔG_{max}/e), where ΔG_{max} is the free energy change of the most thermodynamically unfavorable elementary step, i.e., the potential-determining step (PDS).

Table S2 Zero-pint energy correction (E_{ZPE}), entropy contribution (TS, T=298.15 K), total energy (E), and the Gibbs free energy (G) of molecules and adsorbates in this study.

**Species**	**E_{ZPE} (eV)**	**$-TS$ (eV)**	**E (eV)**	**G (eV)**
H_2	0.27	−0.41	-6.77	-6.91
H_2O	0.56	−0.67	−14.22	-14.33
NO*	0.18	-0.09	-380.92	-380.83
NOH*	0.43	-0.10	-382.76	-382.43
N*	0.11	-0.02	-374.82	-374.73
NH*	0.43	-0.02	-379.64	-379.23
NH_2*	0.73	-0.04	-382.45	-381.76
NH_3*	0.95	-0.21	-385.72	-384.98

1. Nørskov, J. K.; Rossmeisl, J.; Logadottir, A.; Lindqvist, L.; Kitchin, J. R.; Bligaard, T.; Jónsson, H., Origin of the Overpotential for Oxygen Reduction at a Fuel-Cell Cathode. *J. Phys. Chem. B* **2004,** *108*, 17886-17892.
